# Treatment patterns, efficacy and toxicity of regorafenib in gastrointestinal stromal tumour patients

**DOI:** 10.1038/s41598-017-09132-1

**Published:** 2017-08-25

**Authors:** Gustavo Schvartsman, Michael J. Wagner, Behrang Amini, Chrystia M. Zobniw, Van Anh Trinh, Andrea G. Barbo, Heather Y. Lin, Wei-Lien Wang, Anthony Paul Conley, Vinod Ravi, Dejka M. Araujo, Maria Alejandra Zarzour, Robert S. Benjamin, Shreyaskumar Patel, Neeta Somaiah

**Affiliations:** 1 Division of Cancer Medicine, UT MD Anderson Cancer Center, Houston, TX 77030 USA; 2 Department of Pharmacy Clinical Programs, UT MD Anderson Cancer Center, Houston, TX 77030 USA; 3 Department of Biostatistics, UT MD Anderson Cancer Center, Houston, TX 77030 USA; 4 Department of Radiology, UT MD Anderson Cancer Center, Houston, TX 77030 USA; 5 Department of Pathology, UT MD Anderson Cancer Center, Houston, TX 77030 USA; 6 Department of Sarcoma Medical Oncology, UT MD Anderson Cancer Center, Houston, TX 77030 USA

## Abstract

Regorafenib was approved as third-line therapy for advanced Gastrointestinal Stromal Tumour (GIST) at a starting dose of 160 mg daily 3 weeks on, 1 week off, based on improvement in progression free survival over placebo (4.8 vs. 0.9 months), but the response rate was low at 4.5%. Given the high toxicity rate in GIST patients, there is variability in the post-marketing dosing of regorafenib. We aimed to summarize our experience regarding prescribing patterns, efficacy and toxicity of regorafenib and determine the role of response assessment by Choi criteria in GIST patients. We included 28 patients who received regorafenib from our pharmacy. Baseline patient characteristics and treatment outcomes were recorded and an independent radiologist assessed response using Choi and RECIST. Seventy-nine percent of patients started at a 120 mg continuous daily dosing schedule, different from the standard intermittent dosing schedule. Grade 3/4 adverse events were experienced by 43% of patients. Median progression-free survival was 8.7 months. Continuous dosing with regorafenib at 120 mg daily is the preferred prescribing pattern and appears to be better tolerated and with comparable efficacy to the current standard dose. Similar to imatinib, the partial response rate for regorafenib by Choi (29%) was higher compared to RECIST (4%).

## Introduction

Gastrointestinal stromal tumours (GIST) are a rare mesenchymal tumour that represent less than 1% of GI cancers overall, but are the most common mesenchymal neoplasm of the GI tract with an incidence of around 5,000 new cases per year in the United States. The majority of cases (60%) arise in the stomach, 30% arise in the small bowel and less frequently, they can arise in the appendix, colon, rectum, or oesophagus^1^. Though majority patients have localized disease at presentation, 20% of patients present with unresectable or metastatic disease^2–5^. Surgical resection is the mainstay of curative treatment in early-stage disease, but 40% of resected tumours recur and metastasize^6^. Risk of recurrence is based on size, location and mitotic rate of the tumour.

Targeted therapy with imatinib, a selective tyrosine-kinase inhibitor of KIT, PDGFRA, and ABL, revolutionized treatment and was approved for first-line treatment for advanced GIST in 2002 and for adjuvant treatment in high-risk patients in 2008^7–9^. Although more than 80% of patients treated with imatinib will respond to therapy, half of those responding eventually progress after 2 years on therapy due to acquired imatinib resistance. Sunitinib, targeting vascular endothelial growth factor receptor 1 (VEGFR1), VEGFR2, and VEGFR3, Fms-like tyrosine kinase-3 (FLT3), and the receptor encoded by the proto-oncogene RET, was approved as second line therapy in 2006 after showing improved time to progression compared with placebo in GIST patients previously treated with imatinib^10^.

Regorafenib is a multikinase inhibitor with activity against KIT, RET, RAF1, BRAF, PDGFR, FGFR EGFR1–3 and TEK, approved in 2013 for the treatment of patients with locally advanced, unresectable, or metastatic GIST previously treated with imatinib and sunitinib. In 2012, the initial phase II trial of regorafenib in GIST reported a partial response rate of 12% and stable disease (≥16 weeks) of 67%^11^. Based on the starting dose of 160 mg daily 3 weeks on, 1 week off, 82% required a dose reduction for drug-induced toxicity. Regorafenib was eventually approved at this dosing schedule for GIST based on the results of the phase III study, which demonstrated a median progression free survival (PFS) of 4.8 months with regorafenib compared to 0.9 months with placebo (HR 0·27, 95% CI 0·19–0·39; p < 0·0001)^12^. In this trial, 98% of patients assigned to regorafenib had drug-related adverse events (AEs) and 72% of these patients in the regorafenib group required dose modifications, even with limiting accrual to patients with Eastern Cooperative Oncology Group (ECOG) performance status of 0 or 1. The most common grade 3 events were hypertension (23%), hand-foot syndrome (20%), and diarrhoea (5%).

Due to the high rate of adverse events, toxicity-related dose reductions, and the preference for continuous daily dosing of tyrosine kinase inhibitors (TKIs) in the treatment of GIST, many sarcoma experts start patients on 120 mg continuous daily dosing. Continuous daily dosing is standard with imatinib and also frequently used with sunitinib for the treatment of GIST^13^. This practice stems from the experience in GIST patients, where clinical progression and progression on positron emission tomography (PET) was noted during the week off.

Herein we summarize our post marketing experience with regorafenib in GIST patients and report the efficacy and toxicity of the continuous dosing schedule. We also examine the role of two imaging response assessment criteria frequently used in GIST in this patient cohort. The Response Evaluation Criteria in Solid Tumours, version 1.1^14^ (RECIST) is currently the standard tumour response criteria for most solid tumour assessments in clinical trials. Choi criteria have been proposed as a method that takes into consideration both tumour size and tumour density by computerized tomography (CT) scan, and is critical while assessing response of imatinib in GIST, as responding tumours often become cystic and occasionally increase in size^15, 16^. Choi criteria are more sensitive to detect responses to imatinib and may have prognostic value as compared to RECIST in the first-line setting. Increased responses have also been reported by Choi with sunitinib and regorafenib^17, 18^, but its impact on guiding clinical decisions is not clear beyond first-line therapy.

## Results

### Patient Characteristics and Mutation Profile

We screened 176 patients with GIST treated at The University of Texas MD Anderson Cancer Center (MDACC) during the study period. Of those, 141 were previously treated with a TKI, and 28 patients were identified as receiving regorafenib through the MDACC pharmacy and were included in this study, in order to accurately document the dosing schedule received. Patient characteristics are listed in Table 1. The median age when starting regorafenib was 58 years (range: 21–84). There was a slight male predominance (n = 17, 61%). Among the 27 patients who had mutation results, 85% (n = 23) had a KIT mutation, 4% (n = 1) had PDGFR mutation and 11% (n = 3) were wild-type for both. Seventeen out of the 23 patients with a KIT mutation had an exon 11 abnormality and one had a 6 base-pair insertion on exon 9, while 5 did not have data on which KIT mutation was present. Of the 6 patients who had a second biopsy/resection prior to regorafenib, 5 had a secondary KIT mutation in exon 17 and 1 in exon 13. All patients had previously progressed on imatinib and sunitinib. Notably, 29% of patients had received three or more lines of therapy prior to regorafenib.

**Table 1:** Patient characteristics.

Baseline characteristics	N = 28 n (%)
Age at therapy start, mean (SD)	58 (14.7)
Sex (%)	
— Males	17 (60.7)
— Females	11 (39.3)
Histology (%)	
— Epithelioid	4 (14.3)
— Spindle Cell	14 (50.0)
— Both	2 (7.1)
— Not reported	8 (28.6)
Primary site (%)	
— Stomach	16 (57.1)
— Small Bowel	11 (39.3)
— Oesophageal	1 (3.6)
Site of metastasis (%)	
— Liver	24 (85.7)
— Lung	4 (14.3)
— Peritoneum	15 (53.6)
— Spleen	3 (10.7)
— Bone	5 (17.9)
— Mediastinum	1 (3.6)
— Pelvis	2 (7.1)
— Spine	1 (3.6)
— Retroperitoneum	1 (3.6)
Mutation profile (%)	
— KIT	23 (82.1)
— PDGFR	1 (3.6)
— WT	3 (10.7)
— Not done	1 (3.6)

### Treatment Schedule

Twenty-two patients (79%) were started on 120 mg continuous daily dosing schedule. Six patients (21%) were initiated on standard intermittent dosing. Median duration of treatment was 7.3 months (range, 0.9–18.8 months) among the 26 patients who discontinued therapy during the follow-up period and the median tolerated dose was 120 mg once daily in a continuous dosing fashion (mean daily dose = 103.2 mg; standard deviation (SDev) = 23.5 mg).

### Survival and Response Rate

**Figure 1:**
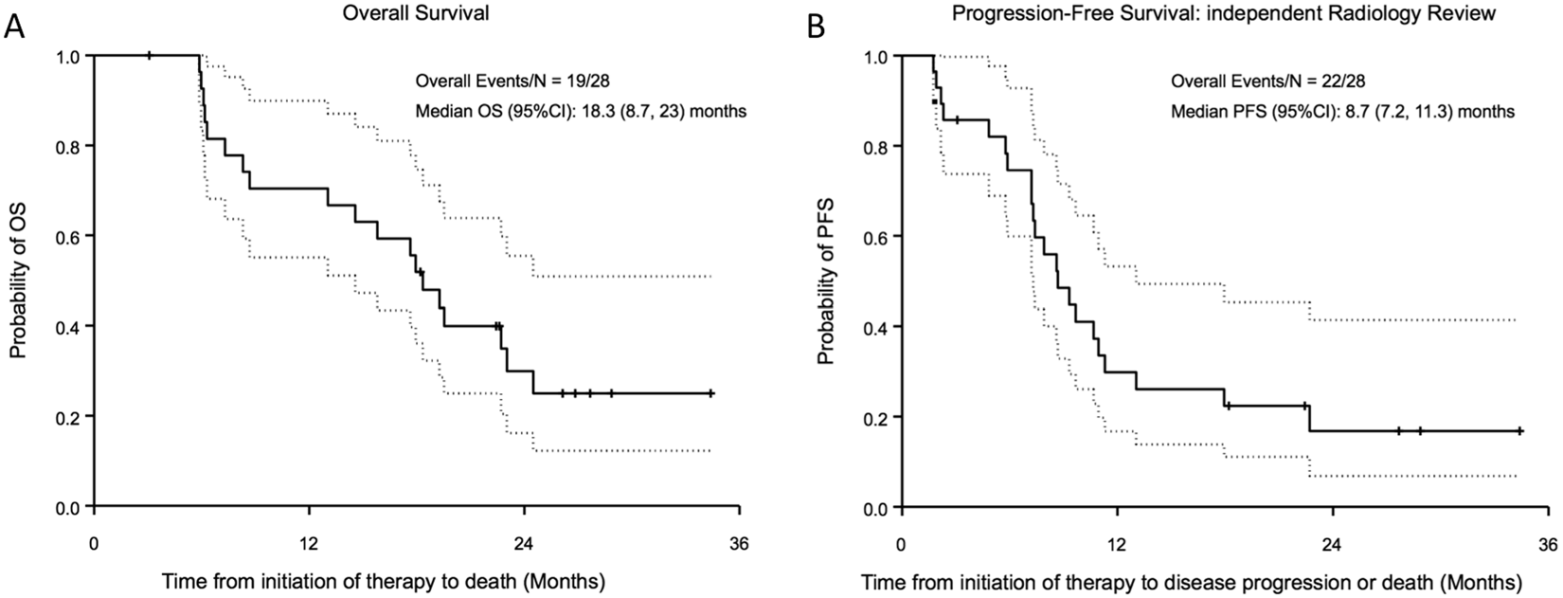
Overall survival and progression-free survival from start of therapy.

The median follow-up time for all patients was 26.8 months (95% CI: 22.4–28.9 months). Nineteen (68%) patients died within the study period and median overall survival (OS) after initiation of therapy was 18.3 months (95% CI: 8.7–23.0 months; Fig. 1A). Twenty-five out of the 28 patients (89%) either progressed or died as assessed by the treating physician and the median progression-free survival (PFS) after initiation of therapy was 9.4 months (95% CI: 5.1–11.8 months). Median PFS based on RECIST as assessed by an independent radiologist was 8.7 months (95% CI: 7.2–11.3 months; Fig. 1B).

Response to treatment was noted in 39% of patients and stable disease was noted in 32% per treating physicians’ assessment based on their review of radiology imaging and reports, which guided clinical decisions. Independent radiology review showed that best overall response rate (ORR) by RECIST (complete response (CR) + partial response (PR)) was 4% (n = 1, PR; time to response: 4 months), while the response at first follow-up at 2 months was 0. Disease control rate (DCR) was 71.4% (n = 20). ORR by Choi was 29% (n = 8, all PRs) and DCR by Choi was also 71.4% (n = 20). None of the patients had a CR. The single responder by RECIST harbored an exon 11 KIT mutation, compared to 5 out of the 8 responders by Choi with the same mutation. Two of the remaining 3 Choi responders were wild-type and one had an exon 18 PDGFR mutation. Response rates are summarized in Table 2.

**Table 2:** Summary of median OS and PFS by RECIST 1.1 and Choi at first follow-up – landmark analysis

Response at first follow-up	Median OS in months by RECIST 1.1		Median PFS in months by RECIST 1.1		Median OS in months by Choi		Median PFS in months by Choi	
	Estimate	95% CI	Estimate	95% CI	Estimate	95% CI	Estimate	95% CI
PD	4.8	(2.6, 13.4)	1.3	(0, 4.8)	3.9	(2.6, 21.2)	2.6	(0, 7.8)
SD	20.6	(15.5, not reached)	8.2	(6.2, 20.6)	15.5	(5.1, 22.2)	9.0	(0, not reached)
PR	N/A**		N/A**		17.7	(5.1, not reached)	6.8	(4.0, 8.2)
p-value*	0.005		<0.001		0.266		0.112	

^*^From log-rank test. PD, progressive disease; SD, stable disease; PR, partial response; OS, overall survival; PFS, progression-free survival; CI, confidence interval.

^**^One patient had a partial response by RECIST and was excluded from analysis.

**Figure 2:**
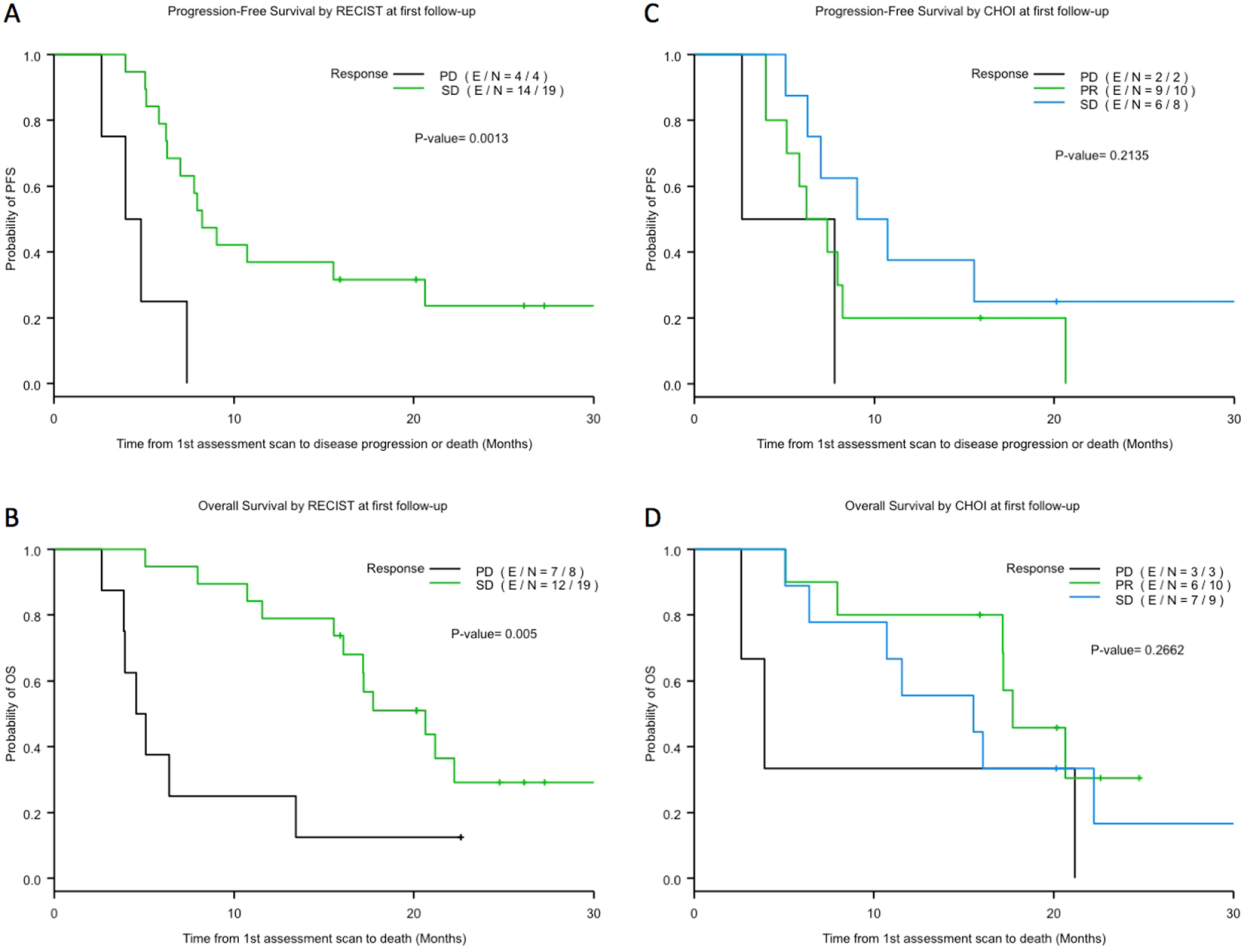
Comparison of progression-free survival and overall survival among patients with PR, SD and PD by RECIST 1.1 (**A** and **B**) and Choi criteria (**C** and **D**). Only one patient had a PR by RECIST 1.1 and hence was excluded from analysis (**A** and **B**).

As demonstrated in Fig. 2A,B, patients with stable disease (SD) had a significantly longer PFS and OS compared to patients with progressive disease (PD) using RECIST at first follow-up for landmark analysis (p =  < 0.01). By Choi criteria, there was a non-significant trend towards improved PFS and OS for patients with PR or SD compared to patients with PD (Fig. 2C,D). No significant difference in OS and PFS was observed among those who had a PR by Choi and had SD by RECIST when compared to those who had stable disease by both criteria.

### Toxicity

Twenty-six of the 28 patients (93%) experienced any grade AEs, while 12 (43%) experienced grade 3/4 AEs (Table 3). The most common all-grade AEs observed were hand-foot syndrome (61%), fatigue (50%) and weight loss (43%). The most common grade 3/4 AEs were hand-foot syndrome and fatigue (18% each). Treatment interruptions were noted in 43% of patients. Dose reductions were required in 61% (n = 17) of all patients (4 out of 6 (66%) patients receiving intermittent dosing schedule, and 13 out of 22 (59%) patients receiving continuous schedule). Adverse events that led to discontinuation of therapy occurred in 6 (21%) patients (3 out of 6 50%) on the intermittent dosing schedule, and 3 out of 22 (14%) on the continuous dosing schedule. The median dose prior to discontinuation was 80 mg (mean dose = 97.9 mg, SDev = 25.1 mg).

**Table 3:** Treatment-related adverse events.

	All grades N = 28 n (%)	Grades 3/4 N = 28 n (%)
Any event	26 (92.9)	12 (42.9)
Hand-foot skin reaction	17 (60.7)	5 (17.9)
Fatigue	14 (50.0)	5 (17.9)
Weight loss	12 (42.9)	4 (14.3)
Nausea	7 (25.0)	2 (7.1)
Diarrhoea	11 (39.3)	2 (7.1)
Hypertension	7 (25.0)	2 (7.1)
Neuropathy	2 (7.1)	2 (7.1)
Arthralgia	1 (3.6)	0
Creatinine elevation	1 (3.6)	0
Liver function tests elevation	1 (3.6)	0

## Discussion

Regorafenib is currently approved for the treatment of patients with locally advanced, unresectable, or metastatic GIST, as third-line therapy after imatinib and sunitinib. Based on the phase 1 study, the maximum tolerated dose for further evaluation was determined to be 160 mg, on a 3-week on, 1-week off fashion^19^. Following the efficacy noted in the phase II trial, the phase III trial (GRID), using the same regimen, showed an improved PFS compared to placebo, leading to its approval 2013^20^. In the real world, however, some GIST experts tend to favour continuous dosing of TKI in patients with GIST, given the rapid progression noted when these patients are taken off their targeted therapy. This report is the first review regarding continuous dosing of regorafenib in GIST patients.

The GRID study revealed a relatively high rate of toxicity for the starting dose of 160 mg, on a 3-week on, 1-week off schedule. Grade 3/4 AEs occurred in 61% of cases and nearly all patients (98%) assigned to regorafenib had some drug-related adverse event. In addition, 72% of the patients in the regorafenib group required dose modifications. Median dose of regorafenib was 146.8 mg versus (vs.) 160 mg for placebo recipients during the 3 weeks on treatment. Median PFS was 4.8 months in the regorafenib group vs. 0.9 months in the placebo group (hazard ratio (HR) 0.27, p < 0.0001). No significant difference was seen in OS (22% of events in the regorafenib group vs. 26% in the placebo group), as patients were allowed to cross over to regorafenib upon progression. Six of the 133 patients in the regorafenib group had a PR (4.5%) and SD was noted in 71.4% of patients in the regorafenib group.

Our retrospective review reveals that continuous dosing with regorafenib 120 mg daily is the preferred starting schedule for GIST patients at MDACC. This alternate dosing still resulted in a fair number of toxicities requiring dose reductions. The toxicity profile however, compared favorably (incidence of grade 3/4 toxicity 43% vs. 61%), with a lower rate of dose modifications (61% vs. 72%), and longer treatment duration (7.3 months vs. 22.9 weeks) when compared to patients enrolled on the GRID study. It also showed a comparable efficacy profile (median PFS of 8.7 months vs. 4.8 months, ORR 4% vs. 4.5% and DCR 71.4% vs. 52.6%). The longer PFS could be explained by the fact that a larger percentage of patients had received three lines of therapy or more in the GRID study (43% vs. 29%). Additionally, the longer period on treatment with continuous dosing may have contributed as well. Outside of a clinical trial, little data is available regarding the real-life efficacy of regorafenib. One study using the same dosing schedule as the GRID study reported inferior results as well, with a DCR of only 44%, no responses and a PFS of 4.5 months^21^. Of course, such comparisons are fraught with problems, and our study has the inherent biases of a retrospective, single-institution review with a small number of patients (n = 28). Although the numbers were low in our study, half the patients receiving intermittent dosing schedule (3/6) were discontinued due to toxicity, compared to 14% (3/22) of those receiving continuous dose. Nevertheless, only a prospective trial comparing the two dosing methods will be able to definitively answer the question of non-inferiority of efficacy and toxicity.

The continuous dosing schedule presented in this study, is also supported by the phase 1 pharmacokinetics study, that showed that all doses from 120 mg and above led to a drop in tumour perfusion by 40%, indicating anti-angiogenic effect^19^. With intermittent dosing, plasma levels of VEGF remained high during the 21-day period and dropped to baseline during the 7-day off period. In light of the findings that efficacy in advanced tumours being similar with doses between 120 mg and 220 mg starting doses, and toxicity profile being improved with lower doses, many experts use alternate dosing strategies to improve tolerance and compliance to regorafenib. Some of the alternate dosing strategies used include starting regorafenib at 80 mg and escalating up to 160 mg, 3 weeks on, 1 week off, based on tolerance; or starting regorafenib at a continuous daily dosing at 120 mg (or 80 mg and titrating up to 120 mg), thus avoiding the oscillation in the drug levels, which could theoretically diminish its efficacy. In addition, pro-active monitoring and support for the common toxicities, such as hand-foot syndrome and hypertension, has helped limit grade 3 or higher toxicities in the routine care of GIST patients on regorafenib allowing them to continue regorafenib at the optimal dose more often^22^.

As previously well documented with imatinib, we also found that the Choi criteria were more sensitive than RECIST to detect responses^15, 16^. However, in this population, patients who were deemed stable by RECIST but had a PR by Choi were not shown to have significantly improved OS when compared to those patients with stable disease by both criteria. Of note, in the third-line setting and beyond the response duration is much shorter than with imatinib in the front-line. Thus, in the third line setting, as long as patients are continued on regorafenib if the disease is stable, their outcomes were not significantly different in this small study. This lower response rate and duration of response in third line therapy and beyond is likely secondary to tumor heterogeneity and multiple different resistance mutations, which might also account for this lower Choi response rate. RECIST showed better PFS and OS for patients with disease control versus those with disease progression using landmark analysis (Fig. 2A,B), while the same analysis by Choi showed a similar trend but was not statistically significant (Fig. 2C,D). In one patient, a best response of PD by RECIST was actually assessed as a PR by Choi. Since the treating physicians at MDACC frequently look at tumour density while assessing response, this patient was continued on therapy for additional 4 months until he eventually progressed by both criteria. This advocates for the importance of recognizing Choi responses so patients deriving benefit from therapy can stay on it for a longer period.

In conclusion, this study supports the commonly used regimen of regorafenib at 120 mg continuous daily dosing as it is reasonably well tolerated, and appears to have comparable efficacy to the approved dosing schedule of 160 mg daily for 3 weeks, followed by a one-week recovery period. Choi criteria are more sensitive to detect responses than RECIST, but as long as therapy is continued until progression assessed by Choi, patients with PR/SD by RECIST have a similar outcome compared to patients who have a PR by Choi. A larger, multi-center, prospective, randomized study would be required to confirm our findings and to further assess the role of Choi criteria in regorafenib-treated patients. However, the feasibility to do such study is low, as time, expense and patient resources are limited. A pooled, multi-centric retrospective analysis with a control group in GIST-expert centers would be the preferred next step to validate our findings.

## Methods

### Patient selection and data collection

We queried MDACC pharmacy administration database to identify all GIST patients who received regorafenib between March 2013 and October 2014 through our institutional pharmacy. We used this database so we could accurately capture dosing patterns and these patients had all their follow up at MDACC. Electronic clinic records were reviewed and data was retrieved for patient demographics, mutation profile, treatment data, starting dose, dosing schedule, median dose tolerated, toxicity profile according to the National Cancer Institute Common Terminology Criteria for Adverse Events (version 4.0), response rate and survival after initiation of treatment. An independent radiologist reviewed images to assess response by RECIST 1.1 and Choi criteria^14, 16^. This study was compliant per the Institutional Board Review of MDACC.

### Statistical analysis

Descriptive statistics were generated to summarize patient demographics and clinical characteristics. OS (time from initiation of therapy until death) and PFS (time from initiation of therapy until progression or death) were estimated by the Kaplan-Meier method^23^. DCR was defined as PR + SD for at least 12 weeks. Time point of progression was determined based on imaging by the treating physician as noted in medical records and also by an independent radiologist as part of this study. Log-rank test^24^ was performed to test the difference in survival (PFS and OS) between RECIST assessment groups as well as Choi groups. In the said comparison of OS and PFS by RECIST 1.1 and Choi response, landmark analysis was performed in that the survival time was calculated from the date of first follow-up assessment. Kaplan-Meier plots were also generated to describe the survival curves by best response from start date of treatment. Statistical significance was determined using a two-sided p-value < 0.05. All statistical analyses were done using SAS 9.4 (SAS Institute, Inc., Cary, NC).
